# A review of the description of patient withdrawal in trial protocols and patient information sheets (PIS)

**DOI:** 10.1186/1745-6215-16-S2-P46

**Published:** 2015-11-16

**Authors:** Anna Kearney, Anne Daykin, Ali Heawood, Athene Lane, Jane Blazeby, Mike Clarke, Paula Williamson, Carrol Gamble

**Affiliations:** 1 University of Liverpool, Liverpool, UK; 2 University of Bristol, Bristol, UK; 3 Queen's University, Belfast, UK

## Background

Under Good Clinical Practice guidelines, patients have the right to withdraw from a trial at any time, for any reason. However, definitions of withdrawal vary from patients ceasing all trial specific activities to stopping the assigned intervention while continuing with follow up. Early withdrawal with no further follow up is problematic and leads to missing data and research waste.

Health Research Authority guidance states that Patient Information Sheets (PIS) should clearly inform patients what is expected with regards to subsequent follow up and the use of existing data if they withdraw.

## Aims

To assess how withdrawal, retention and the value of outcome data collection is described in PIS.

51 adult or parent PIS from HTA-funded trials starting in 2009-2012 were obtained from protocols, websites or the researchers.

PIS were uploaded into NVivo 10 along with text extracted from the corresponding protocols describing patient withdrawal. Broad a priori categories were used with an iterative analysis process to develop coding categories.

## Results

PIS frequently state that the patient can withdraw at any point without having to give a reason. In contrast, very few inform patients they may be asked to give a withdrawal reason if they are willing to do so or refer to the withdrawal options described in the protocol. We present a review of the PIS statements used along with examples of good practice.

## Conclusions

Withdrawal is poorly described in PIS. Working with patients to address this might improve patient retention in trials, reducing missing data and waste.

